# Adaptation strategies of *Cunninghamia lanceolata* seedlings to light intensity gradients based on morpho-physiological trade-offs

**DOI:** 10.3389/fpls.2025.1646842

**Published:** 2025-09-18

**Authors:** Qingqing Liu, Zhijun Huang, Xianhua Zou, Xiangqing Ma, Bo Liu

**Affiliations:** ^1^ School of Soil and Water Conservation, Jiangxi University of Water Resources and Electric Power, Nanchang, China; ^2^ Jiangxi Provincial Engineering Research Center for Seed Breeding and Utilization of Camphor Trees, Jiangxi University of Water Resources and Electric Power, Nanchang, China; ^3^ College of Forestry, Fujian Agriculture and Forestry University, Fuzhou, China; ^4^ College of Life Sciences, Qufu Normal University, Qufu, China

**Keywords:** plantation, morphological plasticity, physiological adjustments, non-structural carbohydrates, light

## Abstract

**Introduction:**

*Cunninghamia lanceolata* (Lamb.) Hook is a high-quality, fast-growing plantation-tree species widely distributed in southern China, and a commercial timber species unique to China that plays a vital role in meeting wood demand and maintaining ecological security. Although the morphological and physiological adaptations of *C. lanceolata* seedlings to light stress have been extensively documented separately, their interplay remains a critical gap in our knowledge and understanding of plant ecophysiology. Particularly, the synergistic mechanisms between phenotypic adaptations and metabolic regulation remain unclear.

**Methods:**

This study employed 1-year-old, clonal, *C. lanceolata* seedlings as test materials to investigate the coordinated effects of different light intensity gradients (100%, 68%, 27%, 12%, and 5% of full sunlight) on the morphological and physiological responses.

**Results:**

(1) Under decreasing light intensity, seedling height to diameter ratio and specific leaf area were 30.10% and 64.38% greater than of those recorded under 100% light intensity. The observed changes in growth maximized light capture capacity. Further, root growth, root to shoot ratio, and seedling quality index decreased with decreasing light intensity. (2) Leaf non-structural carbohydrate contents decreased significantly, along with key carbohydrate-metabolizing enzyme activities, and leaf carbon∶nitrogen and carbon∶phosphorus ratios. (3) High light intensities increased cytokinin and abscisic acid contents, whereas the lowest (5%) light intensity tested enhanced the accumulation of gibberellin, but had no significant effect on indoleacetic acid content.

**Discussion:**

These results indicate that *C. lanceolata* seedlings used a dual adaptation strategy that combined “photoprotection under high light intensity” with “efficient resource utilization under low light intensity” through coordinated morphological and physiological adjustments. Our study provides a scientific basis for managing nursery light conditions and plantation light environment during early development of *C. lanceolata* seedlings. Specifically, we recommend a 68% light intensity for optimal seedling production.

## 1 Introduction

Light is a critical, highly variable environmental factor that strongly influences plant growth, persistence, and competitive community interactions. Further, as an essential environmental driver of plant growth, light plays key roles in physiological metabolism, and in determining plant development and morphology (Godo et al., 2011; Liu et al., 2020; Khan et al., 2025). In particular, the early seedling stage is a phase of ongoing development characterized by high sensitivity and extreme vulnerability to environmental stress, whereby severe population losses and reduced survival rates may occur at this stage (Nicotra et al., 2010; Jafari et al., 2018). As light is a key factor for replenishment and survival of plant populations, a thorough understanding of the response of young seedlings to a changing light environment is of paramount importance for best management of the composition, structure, and function of future forest ecosystems (Werger et al., 2002).

During the development and succession of forest stands, the light intensity that is incident on young seedlings and trees growing under the older forest canopy is disrupted by a number of variables, including: spatiotemporal variation, the complexity of community structure, light attenuation by the higher canopy, and seedling interaction with neighboring plants, all of which, together, may result in insufficient light reception. Indeed, the growth and survival of young seedlings and trees in a forest are closely dependent on their ability to endure long lasting, low-light (shaded) environments, and their ability to regulate and adapt to unexpected situations, such as sudden increases in light radiation caused by canopy rupture or silvicultural practices. Thus, for instance, *Larix principis-rupprechtii* Mayr. seedlings showed different growth strategies during the recovery stages after moderate thinning intensity (~45%), which allegedly triggered a strategic shift from shade avoidance to shade tolerance (Wei et al., 2025).

As they respond to continuous spatiotemporal variations in the light environment of their surroundings, plants must be able to sense subtle changes in light intensity and initiate the adaptive morphological and physiological responses that are needed for survival (Li and Kubota, 2009; Messier et al., 2010; Albert et al., 2012; Carlson et al., 2016; Dou et al., 2017; Park and Runkle, 2017). Throughout their life cycle, but particularly, during the early developmental stages, plants generally adapt to changing light conditions through various strategies, including primarily: morphological adaptation (Xiao, 2024), adjustment of the photosynthetic mechanism (Verhoeven and Kornkven, 2024; Bag et al., 2025; Khan et al., 2025), and carbon allocation patterns (Yuan, 2023), among others, such as to enhance their competitiveness and capabilities for survival (Laurans et al., 2024; Wei et al., 2025).

In particular, due to its high quality and fast growth, *Cunninghamia lanceolata* (Lamb.) Hook is a widely used plantation species in southern China. However, the sustainability of *C. lanceolata* forests is currently threatened by limited gap-phase regeneration, poor self-regeneration, and niche compression, particularly in conifer-broadleaf mixed forests and high-density plantations. In the case of *C. lanceolata* plantations, conventional management practice leads to a gradual reduction in light availability within the stand with increasing canopy closure. In such scenario, deliberate pruning and thinning, or the emergence of forest gaps and windows owing to natural disasters, can improve light availability near the ground. Under such circumstances, exploration of the adaptive strategies of understory *C. lanceolata* seedlings to continuously varying light conditions is necessary for sustainable plantation management. Specifically, studies on the tolerance of *C. lanceolata* to variations in light intensity during early development are essential for understanding the growth strategies at play during the initial growth stages.

This study examined *C. lanceolata* seedlings to investigate their adaptation mechanisms to different light intensities (100%, 68%, 27%, 12%, and 5%) during early seedling development. Although previous studies have separately documented the morphological and physiological responses of *C. lanceolata* seedlings to light conditions, several critical questions remain unresolved: (1) how does light intensity regulate the growth and morphological plasticity of *C. lanceolata* seedlings? In particular, we analyzed the response patterns of key morphological indicators to a light gradient, including aboveground and belowground traits. (2) How does leaf carbon metabolism respond to light variation? This includes elucidating the changes in non-structural carbohydrates (NSCs), carbohydrate-metabolizing enzyme activities, and C/N/P stoichiometry. (3) How do leaf endogenous-phytohormone levels adjust to a light gradient? Finally, (4) what is the synergistic relationship between the physiological adaptations observed and seedling morphological development?

This study aimed to elucidate the coordinated “morphology-metabolism-hormone” regulatory mechanisms underlying light adaptation, through integrated morphological, physiological, and metabolic analyses. Our study provides a theoretical foundation for optimizing light environment management in nurseries by offering guidance regarding appropriate pruning and thinning intensities to be applied, and by improving high-efficiency cultivation practices in *C. lanceolata* plantations.

## 2 Materials and methods

### 2.1 Experimental design and treatments

A pot experiment was conducted in a flat open area at the Shunchang State-owned Forest Farm, Fujian, China. Five light intensity treatments were included: 100%, 68%, 27%, 12%, and 5% of full sunlight, each with four replicates. Treatments were established using square rigid frames (each side, 1.8 m) covered with black nylon shade cloth of different mesh sizes, except for full sunlight (100% light intensity), in which case, no shade cloth was used. The frames were placed parallel to the daily track of the sun to minimize spatiotemporal variations of solar radiation, and kept at a distance of approximately 15 cm between the shade cloth and the ground.

Light conditions under each treatment were determined daily at 12∶00 h for 7 d, on clear cloudless days using a light meter (HP350; HiPoint, Taiwan, China). Based on the results, light transmittance of each shade net chamber was determined to be 68%, 27%, 12%, and 5% of full sunlight. The ratio of red to far-red light was determined using a handheld spectrometer (Red/Far-Red Sensor; Skye Instruments Ltd., UK) (**Table 1**). The setting of the light intensity gradient was to approximate the light environments at different positions within the plantation, as much as possible, thereby effectively simulating the actual light conditions experienced during early seedling development.

**Table 1:** Light conditions under different treatments.

Light intensity	Illuminance (Lux)	Photosynthetic photon flux density (PPFD, μmol·m^-2^·s^-1^)	Red/Far red ratio
100%	83622.11 ± 2706.44a	1500.40 ± 49.69a	1.30 ± 0.01a
68%	57102.77 ± 2417.55b	1018.99 ± 43.10b	1.23 ± 0.02b
27%	23055.31 ± 1042.60c	411.44 ± 18.56c	1.25 ± 0.02ab
12%	10232.77 ± 680.94d	182.63 ± 12.19d	1.30 ± 0.03a
5%	3884.60 ± 196.76e	69.52 ± 3.60e	1.29 ± 0.03ab

Data are presented as mean ± standard error. Different lowercase letters indicate significant differences among light intensities at *p* < 0.05.

One-year-old clonal *C. lanceolata* seedlings (Yang-061) were purchased from Nanping Yangkou State-owned Forest Farm, Fujian, China. Seedlings were transplanted to larger pots (30 cm inner diameter and 34 cm height) filled with local mountainous red soil to partially mitigate root space constraints. Pots with one seedling each were placed on trays to prevent root extension into nursery soil, ensuring root development was solely controlled by the potting substrate environment. Seedlings were acclimated for one month in a greenhouse prior to the experiments. Then, uniform, well-developed seedlings (n = 120; mean height, 33.14 ± 3.43 cm, and mean root-collar diameter, 4.70 ± 0.57 mm) were selected and randomly divided into five groups. All the seedlings in each group were subjected to the same light intensity gradient with four replicates per treatment, and each replicate included six seedlings. Light intensity treatments began in June 2020. To ensure that the seedlings received similar light irradiation, and to avoid mutual shading between neighboring seedlings, a distance of 40 cm was maintained between neighboring seedlings, and the pots were rotated once a week. The light intensity trial lasted four months. No fertilizer was applied during the trial period; weeds were regularly cleared manually and seedlings were watered two to three times per week.

### 2.2 Measurement of seedling growth and morphological traits

Seedling height, root-collar diameter, and lateral branch length were measured before light treatment initiation (June 2020) and at harvest (October 2020). Monthly relative growth rates (RGR, height and root-collar diameter) were calculated using the formula: RGRh = (lnH_2_ - lnH_1_)/t, and RGRd = (lnD_2_ - lnD_1_)/t; Where, t represents the experimental duration (four months); H and D represent height and root-collar diameter, respectively, and subscripts 1 and 2 indicate initial and final sampling timepoints, respectively. The height to diameter ratio (H∶D) was calculated as the ratio of H_2_ to D_2_. Lateral branch length increment (△BL) was determined by subtracting lateral branch length at the end of the experiment from the initial length.

After completing all physiological measurements, seedlings were harvested and separated into roots, stems, and leaves. All plant parts were placed in paper bags and oven-dried at 105°C for 30 min, and then at 80°C to constant dry weight, after which, the dry mass of each part was weighed. In turn, the root to shoot biomass ratio (R∶S) was calculated as the ratio of root to shoot dry mass. Seedling quality index (QI) was calculated as: QI = Total dry mass/(Height/Root-collar diameter + Stem dry mass/Root dry mass).

At harvest, three seedlings were randomly selected from each shading frame per light intensity level for morphological measurements of leaves and roots, i.e., 12 seedlings were sampled from each different light intensity level. Ten healthy and fully expanded green leaves were randomly sampled from the same leaf position at the same height from each test seedling and placed in an icebox. After leaf removal, pots were thoroughly watered, and roots were carefully excavated, washed with water, and placed in an icebox. The collected leaf and root samples were transported to the laboratory for morphological analysis.

Leaves and whole root systems were scanned using an Expression 10000XL scanner (Epson, Tokyo, Japan) and analyzed using the WinRHIZO image analysis software v.2003e (Regent Instruments, Québec City, QC, Canada) for leaf length (LL), leaf width (LW), leaf area (foliage without petioles, LA), total root length (TRL), root surface area (RSA), and root volume (RV) (Modrzyński et al., 2015; Huang et al., 2021; Liu et al., 2022). All leaf and root materials were oven-dried at 80°C until a constant mass and then weighed. Specific leaf area (SLA) was estimated as the ratio of leaf area to leaf dry mass; finally, root tissue density (RTD) was estimated as the ratio of root dry mass to root volume.

### 2.3 Measurement of leaf physiological traits

One seedling was randomly selected from each treatment, and healthy leaves from the same height were collected for assessing physiological traits. All determinations included four replicates.

#### 2.3.1 Non-structural carbohydrate content

Non-structural carbohydrates (NSCs) are mainly composed of soluble sugar (SS) and starch (S). Both SS and S contents were determined using the anthrone colorimetric method. Leaves at the same position and height were randomly collected from one seedling from each replicate per treatment, cleaned with distilled water, and ground to a powder. Powdered leaf samples (0.2 g fresh weight) were extracted with 5 mL distilled water and placed in a boiling water bath for 30 min. This was repeated twice to ensure complete extraction. After cooling to room temperature and centrifugation, the supernatant was collected in a 25-mL volumetric flask and made up to the mark with distilled water. This solution was used to determine the SS content. Thereafter, the solid residue obtained by SS extraction was dried, and perchloric acid was added to extract S. The absorbance at 630 nm was measured to calculate SS and S contents using a glucose standard curve, and the SS∶S was calculated. Total non-structural carbohydrate (TNC) content was calculated as the sum of SS and S contents (Liu et al., 2020).

#### 2.3.2 Carbohydrate metabolism-related enzyme activities

We analyzed the activities of some of the key enzymes in the Calvin cycle, including ribulose bisphosphate carboxylase/oxygenase (RubisCO, the rate-limiting enzyme in C_3_ photosynthesis) and fructose-1, 6-bisphosphatase (FBPase), which plays key regulatory role in the cycle, controlling starch/sucrose synthesis, and sugar metabolism-related enzymes, including sucrose phosphate synthase (SPS, key enzyme regulating sucrose biosynthesis and photoassimilate partitioning), adenosine diphosphate glucose pyrophosphorylase (AGPase, the rate-limiting enzyme in starch biosynthesis), and phosphoenolpyruvate carboxylase (PEPC, links glycolysis and the Tricarboxylic Acid cycle, and coordinates C metabolism with cellular energy demands). Enzyme activities were measured using commercial kits (Suzhou Comin Biotechnology, Suzhou, China). Fresh leaf samples (0.1 g) were ground into a fine powder and homogenized in liquid N_2_ using a pre-chilled mortar and pestle. After halogenation, samples were extracted with 1 mL extraction buffer in 2-mL test tubes and centrifuged. Enzymatic reactions were performed according to manufacturer instructions. Absorbance at 340 nm was measured using a Microplate Absorbance Reader (BioTek ELX800; BioTek Instruments Inc., Winooski, VT, USA), and enzyme activity was calculated according to the formula provided in the instructions manual.

#### 2.3.3 C/N/P stoichiometry

Dried seedling root, stem, and leaf samples were ground and sieved through a 1-mm mesh for chemical analysis. Leaf carbon (LC) and nitrogen (LN) contents were measured via dry combustion using an elemental analyzer (VARIO MAX CN; Elementary, Germany). In turn, leaf phosphorus concentration was determined using an inductively coupled plasma optical emission spectrometer (Optima 8000; PerkinElmer Inc., Waltham, MA, USA) after sample digestion with H_2_SO_4_–HClO_4_ solution. Then, concentrations were converted into leaf P (LP) contents. The carbon∶nitrogen (C∶N), carbon∶phosphorous (C∶P), and nitrogen∶phosphorous (N∶P) ratios were calculated as content ratios. All chemical analyses were replicated four times for each light treatment.

#### 2.3.4 Endogenous phytohormone contents

Leaf cytokinin (ZA), abscisic acid (ABA), gibberellin (GA_3_), and indoleacetic acid (IAA) contents were determined by using high performance liquid chromatography (RIGOL L3000; Rigol Technologies, Inc., San Jose, CA, USA).

### 2.4 Statistical analysis

Differences in seedling growth, and morphological and physiological traits across light intensity treatments were analyzed using one-way analysis of variance (ANOVA), followed by Fisher’s Least Significant Difference (LSD) test, when pertinent (*p* < 0.05). All statistical analyses were performed using SPSS v.20.0 for Windows (SPSS Inc., Chicago, IL, USA). Histograms were plotted using Origin v.9.1. Data shown are means ± standard error. Plasticity of seedling growth, morphology, and physiological traits were compared using Plasticity Index (PI), previously described (Huang et al., 2022). The PI for each trait was calculated using the formula: PI = (X_max_ -X̅_min_)/X̅_max_; Where X̅_max_ and X̅_min_ represent the maximum and minimum mean values of each trait across light intensity treatments. The PI values in all treatments were averaged for each trait and then ranked for comparison of relative plasticity.

## 3 Results

### 3.1 Growth and morphological response to light intensity

**Figure 1:**
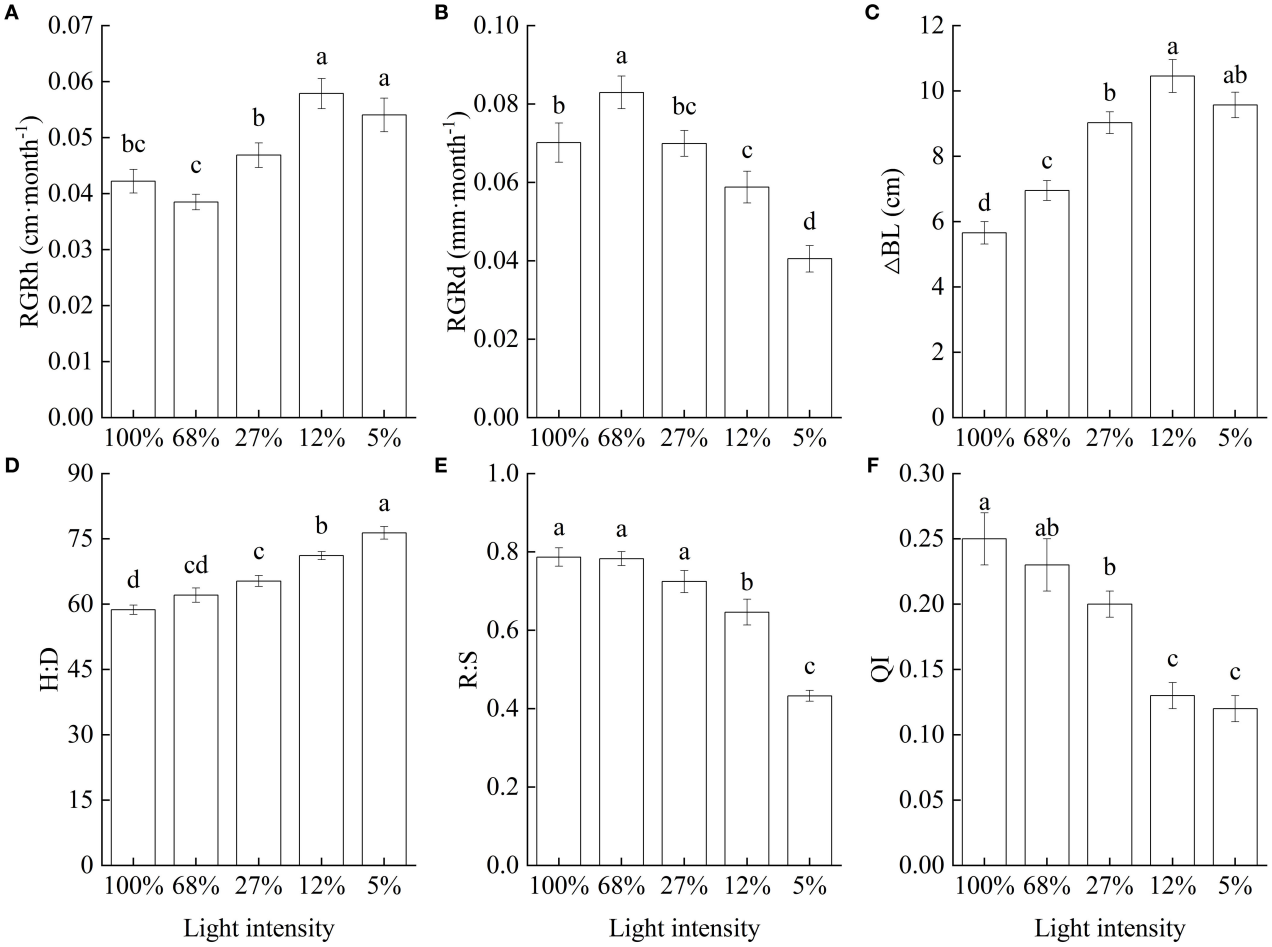
Growth characteristics of *Cunninghamia lanceolata* seedlings under different light intensities. **(A)** Height relative growth rate (RGRh); **(B)** Root-collar diameter relative growth rate (RGRd); **(C)** Lateral branch length increment (△BL); **(D)** Height to diameter ratio (H∶D); **(E)** Root to shoot biomass ratio (R∶S); **(F)** Quality index (QI). Data are presented as the mean ± standard error; different lowercase letters indicate significant differences (*p* < 0.05).

The RGRh significantly increased with decreasing light intensity, peaking at 12% light intensity and followed by a slight decline at 5%, although the difference between the two treatments was not significant. Furthermore, both rate values were significantly higher than those recorded at any other light intensity treatment (**Figure 1A**). In turn, RGRd was highest at 68% light intensity, followed by those at light intensities of 100%, 27%, and 12%, with the minimum rate recorded at 5% light intensity, at only 48.85% of the maximum mean value recorded (**Figure 1B**). Additionally, lateral branch length significantly increased as light intensity decreased, peaking at a light intensity of 12%, at which level, it was 84.97% higher than that recorded at the 100% light intensity treatment, suggesting that low light promoted the elongation of lateral branches in *C. lanceolata* seedlings (**Figure 1C**). Consequently, the H∶D ratio significantly increased with decreasing light intensity, and seedlings showed strongly modified morphological characteristics (**Figure 1D**). In contrast, the R∶S and seedling QI values showed trends opposite to that followed by the H∶D ratio (**Figures 1E, F**).

**Figure 2:**
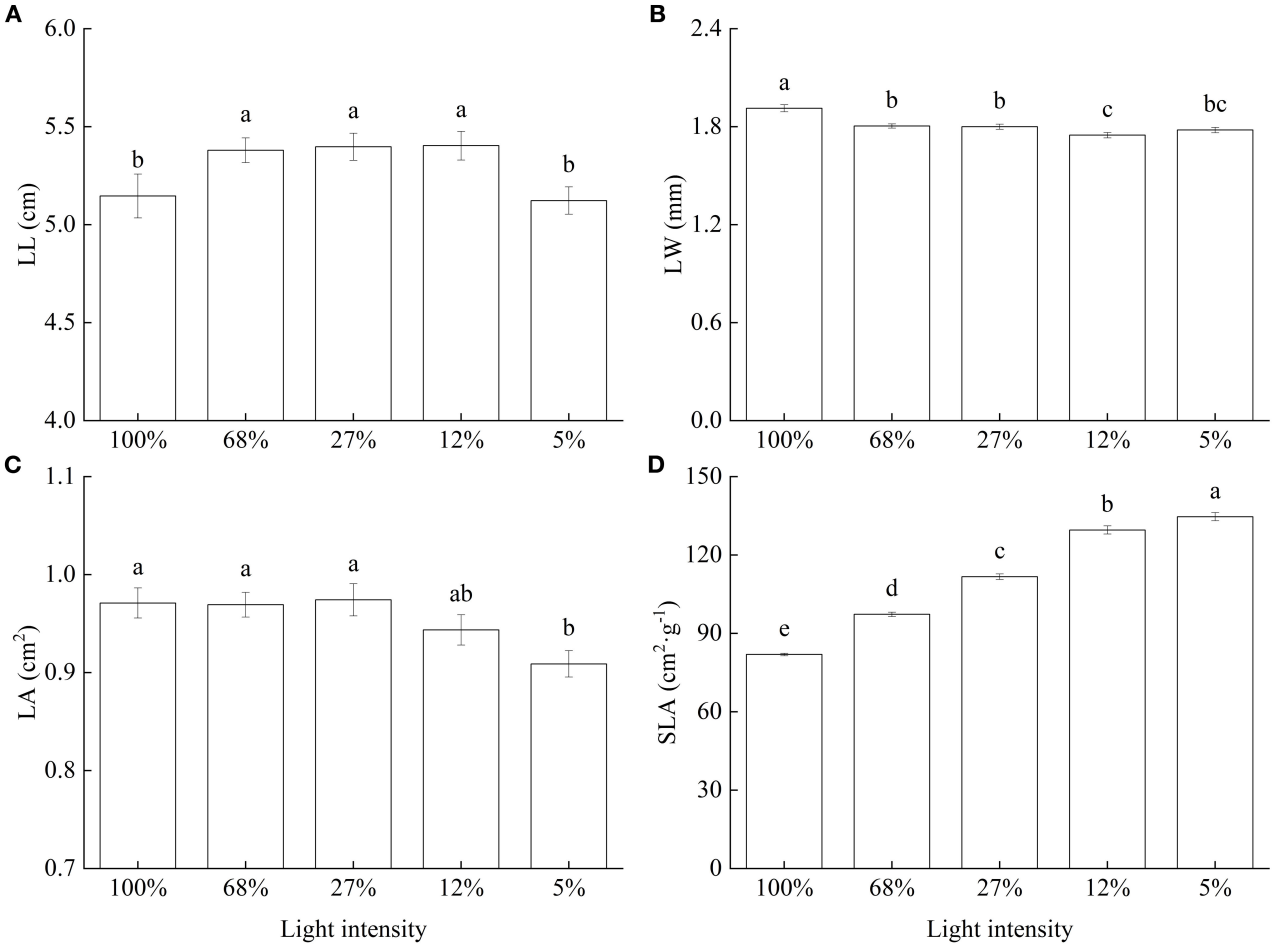
Leaf morphology and specific leaf area of *Cunninghamia lanceolata* seedlings under different light intensities. **(A)** Leaf length (LL); **(B)** Leaf width (LW); **(C)** Leaf area (LA); **(D)** Specific leaf area (SLA). Data are presented as the mean ± standard error; different lowercase letters indicate significant differences (*p* < 0.05).

As for LL, values were generally large and did not significantly differ among the 68%, 27%, and 12% light intensity treatments, whereas they significantly decreased under the 100% and 5% light intensity treatments (**Figure 2A**). Conversely, LW significantly decreased with decreasing light intensity and was highest at 100% light intensity (**Figure 2B**). Meanwhile, LA varied only slightly with light intensity, was lowest at 5% and did not significantly differ from that observed at 12% light intensity (**Figure 2C**). Lastly, SLA significantly increased with decreasing light intensity. In particular, a 64.38% increase in SLA was observed at 5%, relative to that at 100% light intensity (**Figure 2D**).

**Figure 3:**
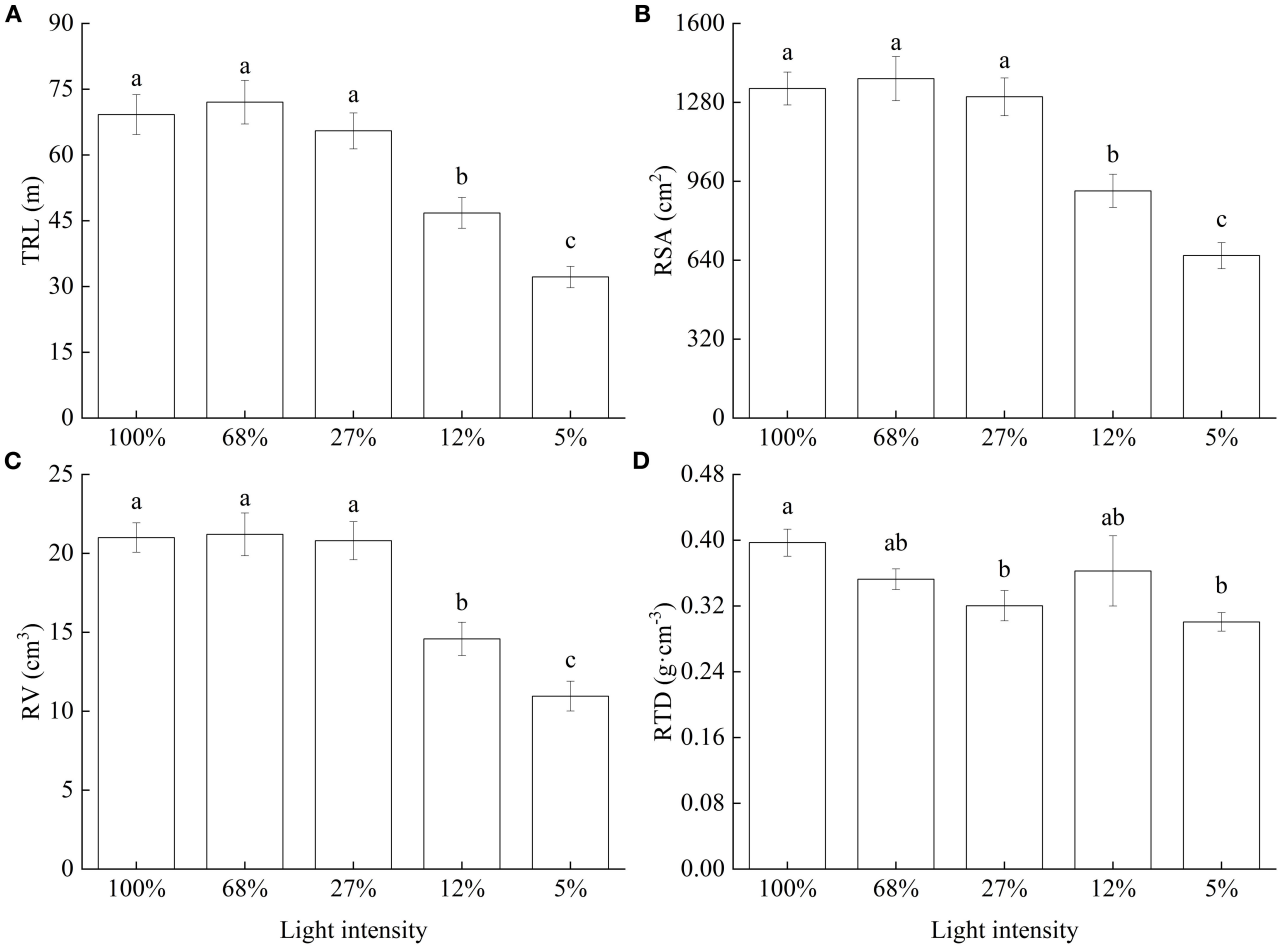
Root morphology of *Cunninghamia lanceolata* seedlings under different light intensities. **(A)** Total root length (TRL); **(B)** Root surface area (RSA); **(C)** Root volume (RV); **(D)** Root tissue density (RTD). Data are presented as the mean ± standard error; different lowercase letters indicate significant differences (*p* < 0.05).

Further, as light intensity decreased, TRL, RSA, and RV all showed consistent trends, i.e., values were relatively large and did not significantly differ among the 100%, 68%, and 27% light intensity treatments; however, values significantly decreased at 12% and were lowest at 5% light intensity (**Figures 3A–C**). Finally, RTD generally decreased with decreasing light intensity (**Figure 3D**).

### 3.2 Leaf carbon-metabolism response to light intensity

**Figure 4:**
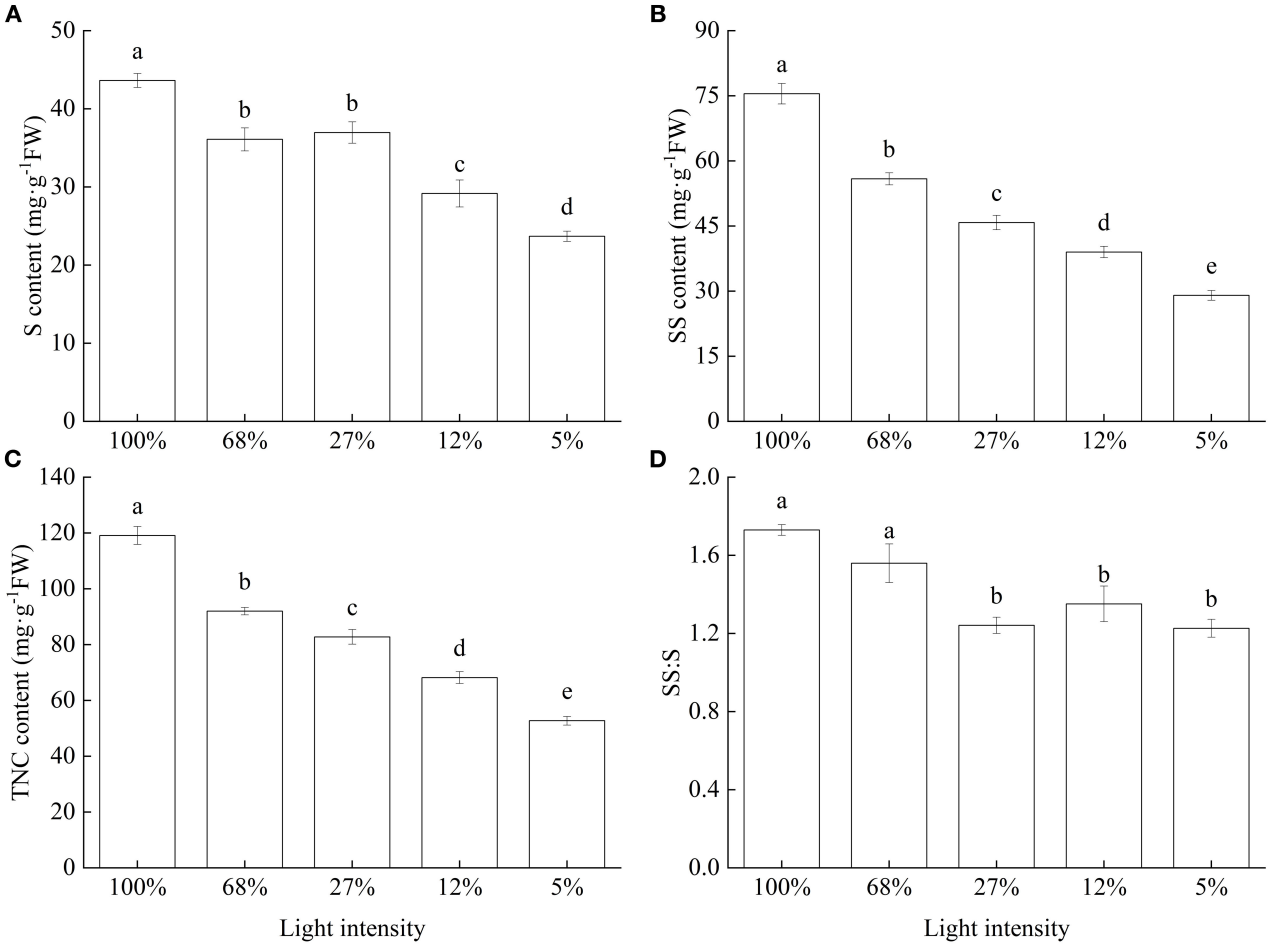
Non-structural carbohydrate contents of *Cunninghamia lanceolata* leaves under different light intensities. **(A)** Starch (S) content; **(B)** Soluble sugar (SS) content; **(C)** Total non-structural carbohydrate (TNC) content; **(D)** Soluble sugar to starch content ratio (S∶SS). Data are presented as the mean ± standard error; different lowercase letters indicate significant differences (*p* < 0.05).

Leaf S, SS, and TNC contents significantly decreased with decreasing light intensity, with the lowest values recorded at 5% light intensity, at which level, they were 54.31%, 38.46%, and 44.27% of those recorded at 100% light intensity, respectively (**Figures 4A–C**). These findings indicate that high light intensity stimulated C accumulation in the leaves of *C. lanceolata* seedlings, increasing TNC contents and its fractions. Further, SS∶S values showed an overall decreasing trend with decreasing light intensity, with high values at 100% and 68% light intensities, and low values at light intensities of 27%, 12%, and 5% (**Figure 4D**).

**Figure 5:**
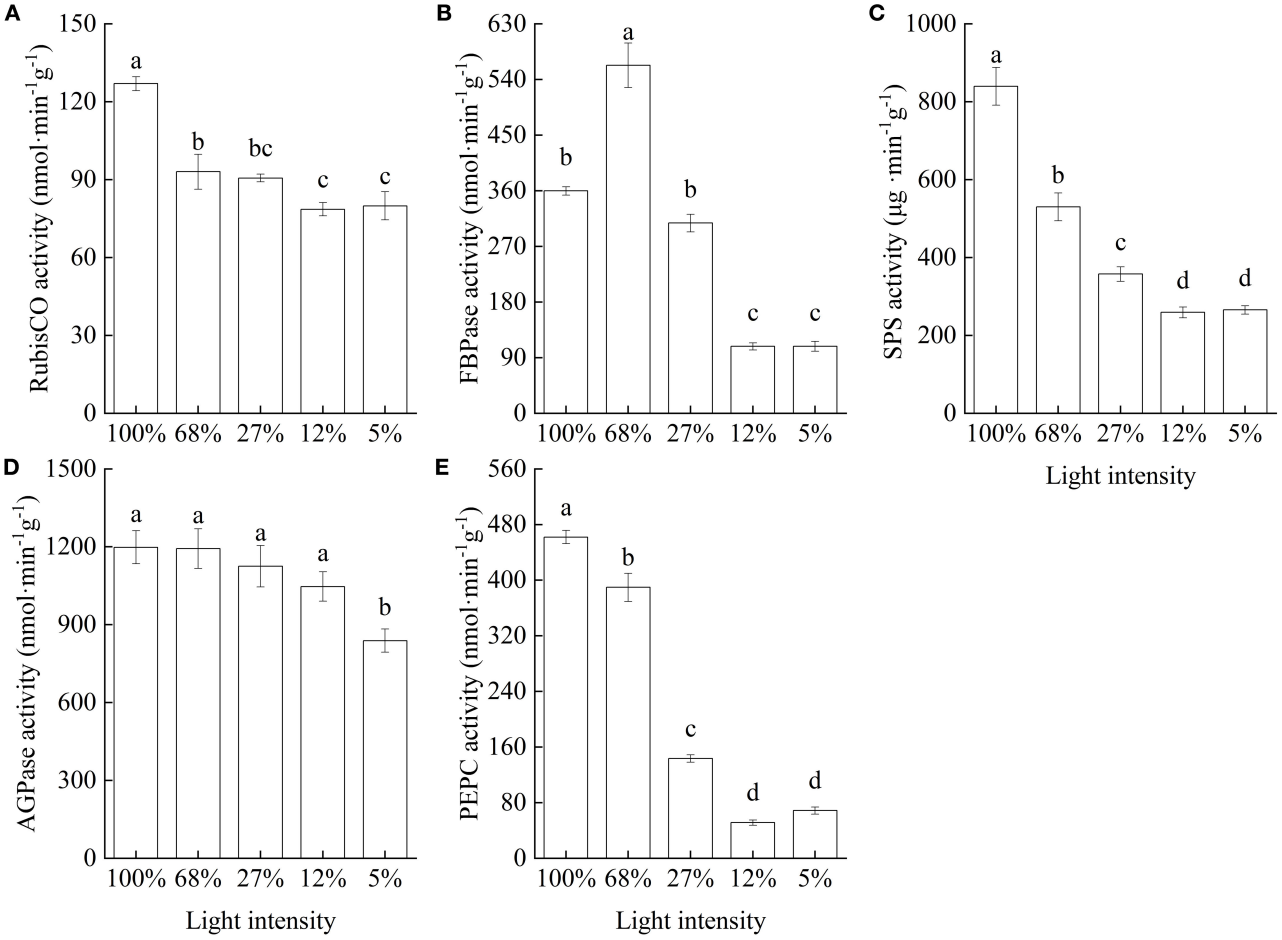
The activities of carbohydrate metabolism-related enzymes of *Cunningham lanceolata* leaves under different light intensities. **(A)** Ribulose bisphosphate carboxylase/oxygenase (RubisCO) activity; **(B)** Fructose-1, 6-bisphosphatase (FBPase) activity; **(C)** Sucrose phosphate synthase (SPS) activity; **(D)** Adenosine diphosphate glucose pyrophosphorylase (AGPase) activity; **(E)** Phosphoenolpyruvate carboxylase (PEPC) activity. Data are presented as the mean ± standard error; different lowercase letters indicate significant differences (*p* < 0.05).

Leaf RubisCO activity significantly decreased with decreasing light intensity (**Figure 5A**). In turn, FBPase activity initially increased significantly and reached the maximum value at 68% light intensity (562.95 ± 35.95 nmol·min^-1^·g^-1^), followed by a significant reduction with further decrease in light intensity until it reached a minimum level at 12% and 5% light intensities, at which levels, FBPase activities were 19.24% and 19.25% of that recorded at 68% light intensity, respectively (**Figure 5B**). Leaf SPS activity significantly decreased with decreasing light intensity. The lowest SPS activities (30.87% and 31.64% of that recorded at 100% light intensity) were recorded at 12% and 5% light intensities, respectively (**Figure 5C**). Consistently, AGPase activity showed an overall decreasing trend with decreasing light intensity, with no significant differences among the values at 100%, 68%, 27%, and 12%, and a significant decrease at the 5% light intensity treatment (**Figure 5D**). Additionally, PEPC activity significantly decreased with decreasing light intensity, with significant decreases of 15.65%, 68.94%, 88.88%, and 85.11% under the 68%, 27%, 12%, and 5% light intensity treatments, respectively, compared to that recorded under the 100% light intensity treatment (**Figure 5E**).

**Figure 6:**
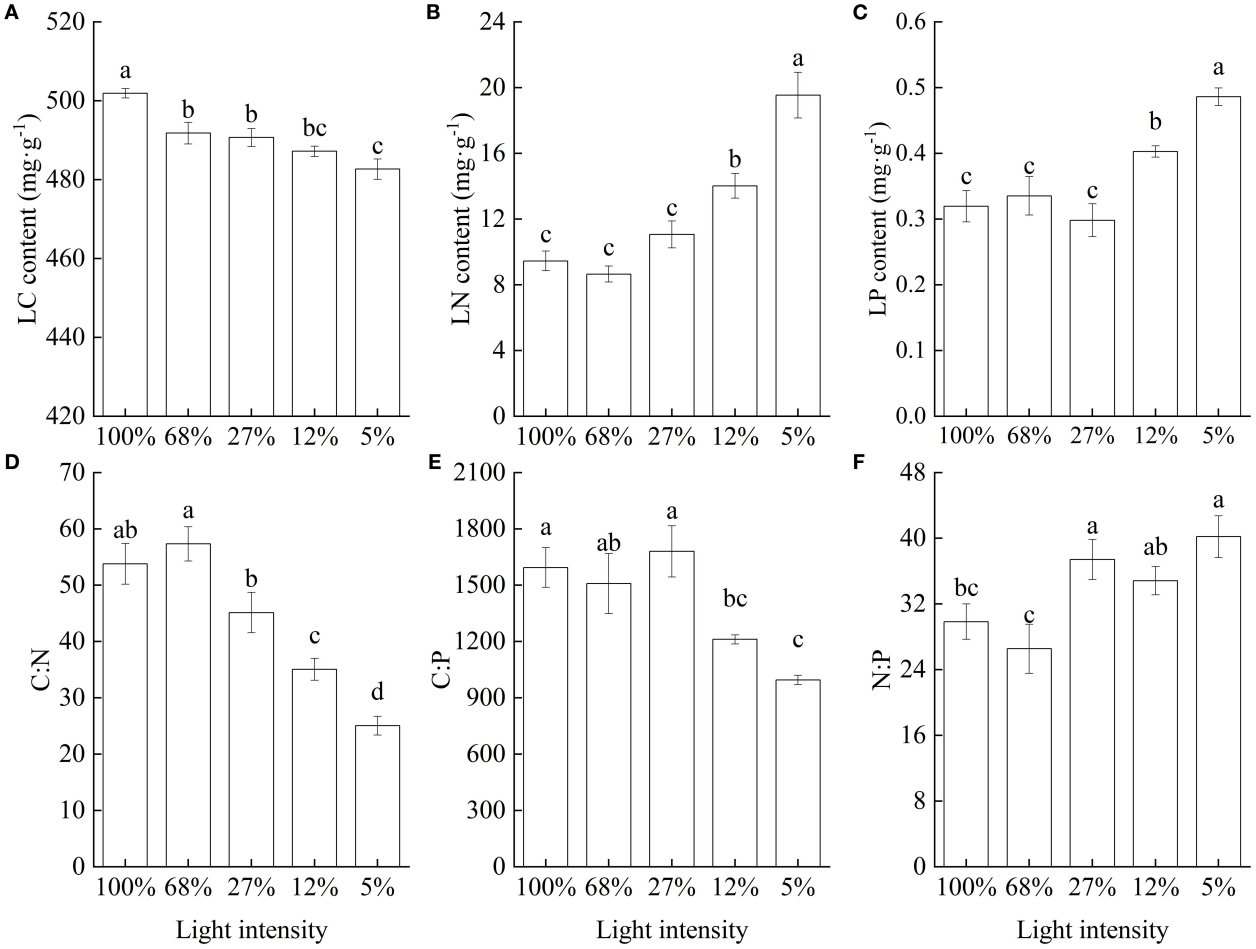
Carbon, nitrogen, and phosphorus contents in *Cunningham lanceolata* leaves under different light intensities. **(A)** Leaf carbon (LC) content; **(B)** Leaf nitrogen (LN) content; **(C)** Leaf phosphorus (LP) content; **(D)** Carbon to nitrogen ratio (C∶N); **(E)** Carbon to phosphorus ratio (C∶P); **(F)** Nitrogen to phosphorus ratio (N∶P). Data are presented as the mean ± standard error; different lowercase letters indicate significant differences (*p* < 0.05).

Leaf C content significantly decreased with decreasing light intensity (**Figure 6A**), whereas both leaf N and P contents significantly increased (**Figures 6B, C**). Consequently, the C∶N ratio showed an overall decreasing trend with decreasing light intensity and was lowest at 5% light intensity, in which case it was only 46.59% of that recorded at 100% light intensity (**Figure 6D**). Meanwhile, the C∶P ratio decreased with decreasing light intensity and reached its minimum value at 5% light intensity (**Figure 6E**). Conversely, the N∶P ratio showed an increasing trend, and the mean values were relatively high at low light intensities (**Figure 6F**).

### 3.3 Response of endogenous phytohormone contents to light intensity

**Figure 7:**
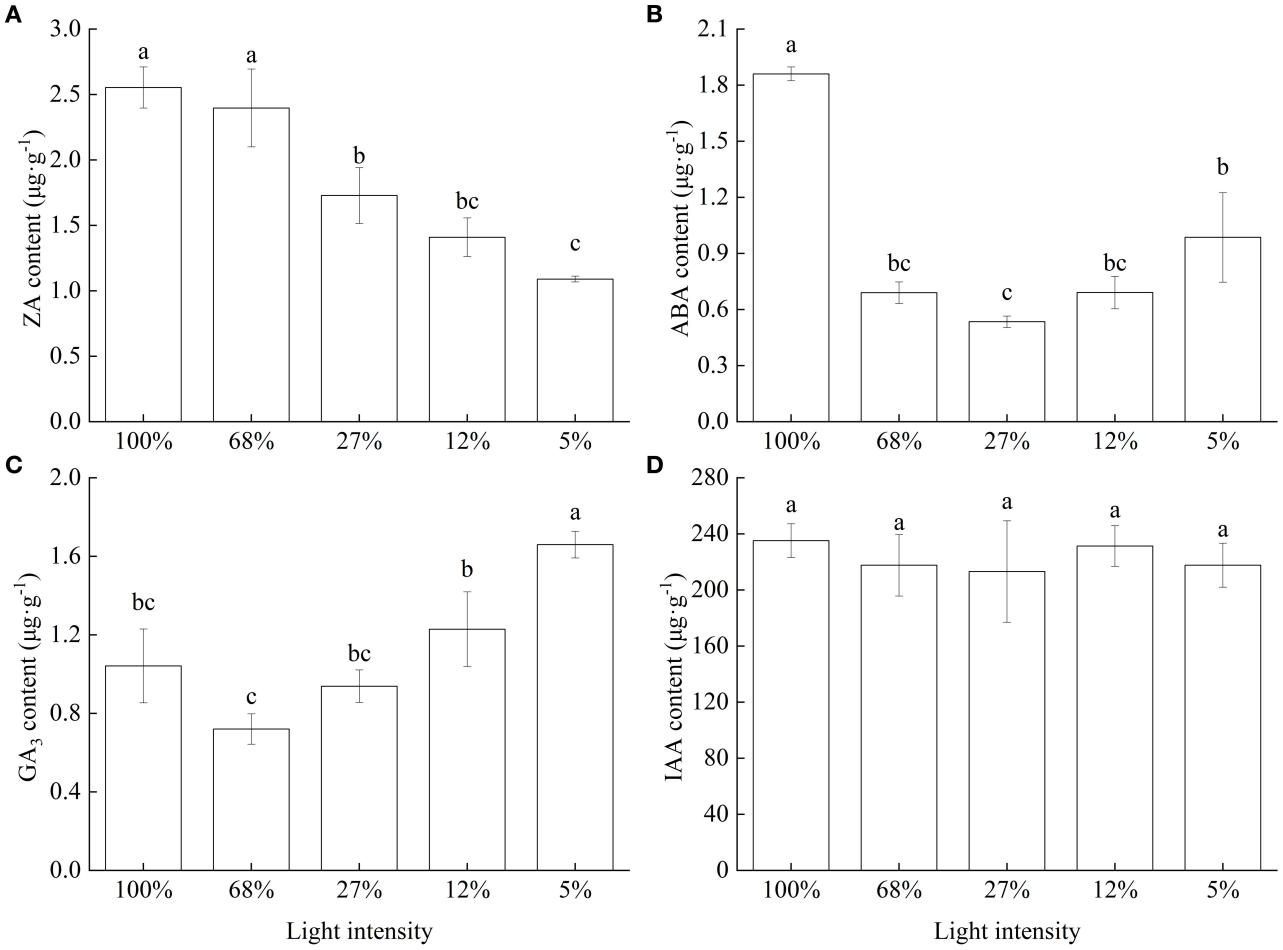
Endogenous phytohormone content of *Cunningham lanceolata* leaves under different light intensities. **(A)** Cytokinin (ZA) content; **(B)** Abscisic acid (ABA) content; **(C)** Gibberellin (GA3) content; **(D)** Indoleacetic acid (IAA) content. Data are presented as the mean ± standard error; different lowercase letters indicate significant differences (*p* < 0.05).

The cytokinin (ZA) content significantly decreased as light intensity decreased, with the lowest content observed at 5% light intensity, at which level, it was 57.34% lower than that recorded at 100% light intensity (**Figure 7A**). In turn, ABA content initially decreased and then increased with decreasing light intensity, with the lowest content observed at 27% light intensity, at which level, it was only 28.73% of that recorded at 100% light intensity (**Figure 7B**). Meanwhile, GA_3_ content initially decreased slightly and then significantly increased, reaching the highest value at 5% light intensity (**Figure 7C**). Finally, although IAA content also decreased with decreasing light intensity, it did not significantly differ (*p* = 0.939) among the different treatment groups (**Figure 7D**).

### 3.4 Estimation of plasticity

The morphological and physiological traits of *C. lanceolata* seedlings showed different plastic responses to light intensity (**Table 2**). In a decreasing order, PI values ranked as follows: carbohydrate metabolism-related enzyme activities > endogenous phytohormone contents > NSC contents > root morphology > seedling growth > mineral nutrient contents > leaf morphology. This ranking indicated that *C. lanceolata* seedlings showed considerable physiological plasticity in response to changes in light intensity. Additionally, the data strongly indicated that the regulation of leaf physiological traits was the primary adaptation strategy of *C. lanceolata* seedlings to changes in light intensity.

**Table 2:** Plasticity indices (PI) for morphological and physiological characteristics in *Cunningham lanceolata* under different light intensities.

Traits	Characteristics	PI	Average PI
Carbohydrate metabolism-related enzyme activities	PEPC activity (nmol·min^-1^·g^-1^)	0.89	0.61
	FBPase activity (nmol·min^-1^·g^-1^)	0.81	
	SPS activity (μg·min^-1^·g^-1^)	0.69	
	RubisCO activity (nmol·min^-1^·g^-1^)	0.38	
	AGPase activity (nmol·min^-1^·g^-1^)	0.30	
Endogenous phytohormone contents	ABA content (μg·g^-1^)	0.71	0.49
	ZA content (μg·g^-1^)	0.57	
	GA_3_ content (μg·g^-1^)	0.57	
	IAA content (μg·g^-1^)	0.09	
NSC contents	SS content (mg·g^-1^)	0.62	0.48
	TNC content (mg·g^-1^)	0.56	
	S content (mg·g^-1^)	0.46	
	SS∶S	0.29	
Root morphology	TRL (m)	0.55	0.45
	RSA (cm^2^)	0.52	
	RV (cm^3^)	0.48	
	RTD (g·cm^-3^)	0.24	
Seedling growth	QI	0.52	0.42
	RGRd (mm·month^-1^)	0.51	
	△BL (cm)	0.46	
	R∶S	0.45	
	RGRh (cm·month^-1^)	0.33	
	H∶D	0.23	
Mineral nutrient contents	C∶N	0.56	0.38
	LN content (mg·g^-1^)	0.56	
	C∶P	0.41	
	LP content (mg·g^-1^)	0.39	
	N∶P	0.34	
	LC content (mg·g^-1^)	0.04	
Leaf morphology	SLA (cm^2^·g^-1^)	0.39	0.15
	LW (mm)	0.09	
	LA (cm^2^)	0.07	
	LL (cm)	0.05	

When PI values of individual characteristics were ranked in descending order, the five most important plasticity characteristics were PEPC activity, FBPase activity, ABA content, SPS activity, and SS content, whereas the five least important plasticity characteristics were LC content, LL, LA, LW, and IAA content. These results indicated strong differences in the PI values of morphological and physiological characteristics with respect to changes in light intensity. By and large, the physiological characteristic that responded most strongly to changes in light intensity was leaf carbohydrate metabolism-related enzyme activity. In contrast, mineral nutrient content showed relatively low plasticity in response to changes in light intensity. Finally, among the growth and morphological characteristics under study herein, root and leaf morphology showed the highest and lowest levels of plasticity, respectively, in response to changes in light intensity.

## 4 Discussion

### 4.1 Morphological responses and phenotypic plasticity

Most plants adapt to shaded environments by regulating their morphological and biomass allocation patterns, such as to facilitate maximum acquisition of light energy and, consequently, enhance photosynthetic efficiency, thereby maintaining normal growth (Liu et al., 2018; Zhi et al., 2021). In particular, plant height and root-collar diameter are basic morphological indicators. When plants perceive low light intensities in the surroundings, a relatively large amount of energy is used for elongation growth, and correspondingly, a relatively low amount of energy is invested in stem radial growth. Such changes in plant growth pattern aims at escaping shading and reaching higher towards the light, thus increasing the amount of light that can be captured by the leaves (Liu et al., 2018). In this study, RGRh and H∶D ratio in *C. lanceolata* seedlings significantly increased as light intensity decreased, whereas RGRd and QI significantly decreased. Under high light intensity (100% and 68%), seedlings showed a dwarf and stocky phenotype, whereas under low light intensity, they showed elongated, thin stems. The threat of limited light to plant survival has led to the evolution of highly plastic adaptive strategies for tolerating or escaping shading under neighboring vegetation (Franklin, 2008). Simultaneously, the increase in lateral branch length increased with decreasing light intensity. This responses to shading were consistent with previous findings (Vandenbussche et al., 2005; Fotis et al., 2018) showing that, when seedlings perceive shade signals, they adopt the shade avoidance response of pioneer species and the resource foraging strategy under low light intensity, manifest as vertical growth, showing morphological adaptations of stem and internode elongation, and the light-foraging strategies of moderately shade-tolerant species, which involve lateral branch growth, i.e., leaves and branches expanding in all directions to increase the range of light and growth space, while avoiding self-shading.

The environmental and evolutionary pressures that shape the development of plant traits may play different roles above and below the ground; therefore, studying the root system is necessary when conducting studies on morphological plasticity. Regulation of the root structure and morphology by plants can directly affect plant ability to access and compete for underground resources, which in turn determines whether the plant has a competitive advantage (Bardgett et al., 2014). Root length determines the depth and breadth of the volume that a plant can explore in the soil. Further, plants with high root volumes and surface areas have a great capacity to absorb water and mineral nutrients (Rajaniemi, 2011). Under high light intensity, seedlings showed higher R∶S values, which means that a relatively large amount of resources was allocated to root growth, and that the TRL RV, RSA, and RTD were relatively large. These morphological characteristics allowed *C. lanceolata* seedlings to efficiently absorb water and nutrients by promoting root growth while increasing root surface area and volume for alleviating the water deficit caused by high transpiration under strong light. Additionally, *C. lanceolata* seedlings may photosynthesize more under strong light, and synthesize and transfer more organic matter to the root system, than under low light conditions. In contrast, under low light intensity, seedlings showed an improved light-harvesting capacity and light use efficiency by increasing biomass allocation to the stem and leaves, SLA, and LN content. The regulation of biomass allocation in *C. lanceolata* seedlings by light intensity was consistent with the prediction of the optimal allocation theory. Specifically, once light becomes a limiting resource, plants allocate a relatively large amount of biomass to the aboveground parts (stems and leaves) to support growth and survival, while reducing the allocation of biomass to the organs (roots) that acquire the non-limiting resource (Liu et al., 2018). The response strategies of *Alnus cremastogyne* (Liu et al., 2013) and *Taxus baccata* (Perrin and Mitchell, 2013) in terms of biomass accumulation and allocation under different light environments are also consistent with the findings reported herein.

Plants perceive light signals through their leaves and convert light energy into chemical energy through the photosynthesis process that takes place within chloroplasts in the leaves. Changes in leaf morphology and structure can reflect the potential of plants to adapt to the light environment and the underlying mechanisms (Carlson et al., 2016). The capacity of plants to adjust leaf morphology (i.e., show high phenotypic plasticity) in disturbed environments is advantageous to resource utilization and growth (Costa et al., 2025). The light adaptation strategy using leaves encompasses efficient light-harvesting mechanisms under low light and photoprotection under high light intensities. Maximizing light capture while minimizing the detrimental effects of high light intensity is a coordinated developmental response (Li et al., 2021). Among all the leaf features, SLA is an important indicator for evaluating the relationship between light acquisition and light utilization strategies in plants. In this study, SLA of *C. lanceolata* seedlings significantly increased with decreasing light intensity, indicating that, low light intensity enhanced the capacity for light interception and capturing scattered light at the expense of relatively small resource inputs by increasing the relative leaf area for light capture, a finding which is similar to others previously reported (Matyssek et al., 2010; Sevillano et al., 2016). In contrast, because high intensity light destroys the structure of photosynthetic tissues, plants must produce relatively small and thick leaves with a reduced SLA under intense light conditions, a phenotype that permits heat dissipation and helps in avoiding the detrimental effects of overheating and high transpiration rates (Nascimento et al., 2015). At 100% and 68% light intensities, *C. lanceolata* seedlings showed relatively low LN content. Reducing N distribution in light-trapping components reduced light energy capture by the leaves to avoid damage to the photosynthetic tissues caused by high light intensities; therefore, this is a protective regulatory mechanism at play in the leaves. In contrast, the increases in SLA and LN content under lower light intensities were favorable for improving the leaf light-trapping capacity and absorption efficiency in the shaded environment.

### 4.2 Light-dependent modulation of leaf carbon metabolism

Carbohydrates, particularly NSCs, the products of photosynthesis including SS and S, are important energy sources for plant respiration, metabolism, and growth (Liu et al., 2020; Guo et al., 2024). On the other hand, SS are important for osmotic adjustment in plants and their accumulation is believed to be an adaptive strategy for maintaining the cellular osmotic pressure of leaves under strong light. Indeed, SS content in *C. lanceolata* leaves was significantly high at 100% light intensity but decreased with decreasing light intensity, consistent with previous observations in a study on *Bletilla striata* (Zhou, 2020). There are numerous factors that influence the accumulation and distribution of NSCs within plants, especially those that have significant effects on photosynthesis-related enzyme activities and photosynthetic performance, and metabolic carbon dynamics. Not surprisingly, one such factor is light intensity (Xie et al., 2018; Liu et al., 2020; Guo et al., 2024). Indeed, darkness or extreme shading trigger sugar starvation in leaf tissues by inhibiting leaf photosynthesis and CO_2_ assimilation (Asad et al., 2024). In this study, leaf S, SS, and TNC contents in *C. lanceolata* seedlings significantly decreased with decreasing light intensity. Consistently, shading significantly reduced SS, S, and NSC contents in the leaves of *Fraxinus mandshurica* (Huo et al., 2009). Overall, NSCs reflect the relationship between C gain (photosynthesis) and loss (respiration and growth). Thus, under low light intensities, all three S, SS, and TNC contents decreased, presumably as a result of seedlings using stored energy for growth, while C fixation declined owing to the above mentioned effects of low light intensity on photosynthesis performance. Similarly, NSC content in shaded leaves of *Quercus aliena* seedlings is reportedly significantly lower than that under natural full sunlight, and seedlings face difficulty maintaining C balance and surviving in an extremely shaded environment (Chen et al., 2017).

The Calvin cycle is the core metabolic pathway for carbon fixation in photosynthesis. Research has confirmed that, as a key enzyme of leaf photosynthetic process, RubisCO plays a fundamental role in the first step of the cycle (Bauwe, 2023). This study showed higher RubisCO and FBPase activities in the leaves of *C. lanceolata* seedlings kept under the higher light intensities tested. In other words, under higher light intensity, highly active RubisCO catalyzes the combination of ribulose-1,5-bisphosphate (RuBP) with CO_2_ to form 3-phosphoglycerate (3-PGA), thus completing the carboxylation reaction of photosynthesis and promoting the reduction of 3-PGA. Concomitantly, FBPase regulates the regeneration of RuBP, which in turn promotes photosynthetic C assimilation and increases the net photosynthetic rate. Additionally, RubisCO activity is closely related to the photochemical efficiency of the photosystem reaction center and usually increases with increasing light intensity, affecting the leaf net photosynthetic rate (Ozawa et al., 2023). Previous studies have shown that low light stress may cause obstruction of photosynthetic electron transport and reduced C assimilation-associated enzymes activities (Wang et al., 2014; Huang, 2024). A case in point, FBPase catalyzes the hydrolysis of fructose-1,6-bisphosphate to yield fructose-6-phosphate and inorganic phosphate, thus playing a key regulatory role in the Calvin cycle and gluconeogenesis pathways, as FBPase activity directly affects the accumulation of carbohydrates and the efficiency of photosynthesis (Khan et al., 2025). In this study, RubisCO, SPS, AGPase, PEPC, and FBPase activities in the leaves of *C. lanceolata* seedlings all decreased with decreasing light intensity. This result suggests that low light treatment adversely affected the activities of carbohydrate-metabolizing enzymes, resulting in a decrease in carbohydrate biosynthesis and metabolism. Specifically, *C. lanceolata* leaves exposed to prolonged light deprivation or low light intensity experienced an inability to conduct C assimilation at a rate above that of respiration, which in turn disrupted energy supply, metabolic biosynthesis, and C balance. Under such conditions, *C. lanceolata* seedlings had only stored reserves to sustain growth, which led to a rapid decline in leaf NSCs levels. These results were validated by our observation that NSC contents significantly decreased with decreasing light intensity.

Light and plant nutrient acquisition are closely related. Indeed, shading can reportedly affect nutrient uptake, transport, and partitioning within plants (Zhi et al., 2021). In this study, exposure to high light intensity led to C gain rates higher than C demand, resulting in NSC storage. In contrast, and in agreement with previous findings (Sun et al., 2020), leaf C content significantly decreased under shade treatment, likely because of reduced photosynthetic efficiency and a subsequent decrease in organic matter. The relatively high C∶N ratio at 100% light intensity indicated that *C. lanceolata* seedlings showed primary C storage coupled with relatively slow growth under strong light conditions, as evidenced by the growth pattern and NSC content variation in response to light intensity. The leaf C∶N ratio significantly decreased with decreasing light intensity, indicating a reduced ability for using nutrients, probably owing to reduced photosynthesis and root growth under low light intensity, which affected nutrient uptake, translocation, and utilization. The energy required for N and P uptake and transport is derived from photosynthesis, which depends on the proteins and enzymes synthesized using N and P during photosynthesis (Tripathi et al., 2019). Under shading conditions, plants increase N and P allocation for enhancing protein/chlorophyll synthesis and sustaining enzyme activities to improve light use efficiency. As a result, plants usually have relatively high N and P contents under low light conditions (Xie et al., 2017; Zhi et al., 2021), and our results were consistent with this pattern: *C. lanceolata* seedlings showed a distinct preference for N and P absorption and utilization under low light. Thus, N and P contents significantly increased with decreasing light intensity, particularly at 5% light intensity, at which level, leaf N content was 2.07 times higher than that at 100% light intensity. However, the strategy of N and P response to decreasing light intensity may be species-specific. For example, *Nicotiana tabacum* showed decreasing N and P contents with decreasing light intensity (Qiao, 2007); meanwhile, N content and N∶P increased, while P content decreased with increasing shade intensity in leaves of *Ulmus elongata* (Luo et al., 2022). The N∶P ratio reflects a critical biochemical constraint in plants, representing the trade-off in resource allocation between N-rich proteins and the P-rich ribosomal RNA used for their production (Sterner and Elser, 2002; Ye et al., 2014). With high diagnostic value for nutrient deficit, the N∶P ratio serves as an important indicator for evaluating the limiting patterns of nutrient supply status of plants in the environments (Tessier and Raynal, 2003; Ågren et al., 2012). Numerous studies have suggested that there is a critical value for the N∶P ratio in plants (Koerselman and Meuleman, 1996; Tessier and Raynal, 2003; Güsewell, 2004). Further, studies in China have shown that, when the N∶P ratio is below 14, plant growth is limited only by N, when it is above 16, growth is mainly limited by P, and when it is between these threshold values, a co-limitation by both N and P prevails (Koerselman and Meuleman, 1996). In this study, N∶P ratio was greater than 16 under the different light treatments, indicating that P was the main growth limiting factor for *C. lanceolata* seedlings. Coincidently, the culture medium used to grow the experimental seedlings in this study was local mountains red soil, which accurately reflected the soil conditions in which *C. lanceolata* grows in its main planting area. Indeed, soil P deficiency is a common problem in red soil areas in southern China.

### 4.3 Phytohormone responses to light intensity

Endogenous phytohormones act as chemical messengers. Their levels affect plant sensitivity to the environment, coordinate growth and development, and plant intercellular communication (Colebrook et al., 2014). Particularly, ABA significantly reduces damage to the ultrastructure of chloroplasts at high temperature and enhances their thermal stability (Hua and Li, 2021). Leaves of *C. lanceolata* seedlings growing under high light intensity showed high ZA and ABA contents. In turn, GA_3_ increases plant height, branch number, xylem development, and secondary stem growth (Bagale et al., 2022; Shah et al., 2023; Zhou et al., 2024; Tang et al., 2025). *C. lanceolata* seedlings grown under shading for a long time commonly showed high GA_3_ contents, which in turn promoted stem elongation and increased the biomass allocation ratio of the aboveground parts; however, it significantly reduces root biomass (Wang et al., 2014). These findings are consistent with our own results, which showed that RGRh of *C. lanceolata* seedlings increased and the R∶S decreased as light intensity was reduced.

## 5 Conclusions

Under high light intensities (100% and 68%), *C. lanceolata* seedlings showed a dwarf, stocky phenotype and adopted protective strategies. Seedlings promote root growth by allocating more resources to the root system, which is characterized by a larger R∶S. This allows them to absorb more water to alleviate the water deficit caused by heat and high transpiration rates. Under high light intensity, *C. lanceolata* seedlings adopted photoprotective strategy to reduce light energy capture by lowering the SLA and LN content, and showed higher ABA content, which can slow down the disruption of chloroplast structure caused by high light intensity and high temperature. Under such circumstances, seedlings adopt a conservative resource-use strategy that favors carbon storage and accumulation of NSCs helping to maintain cellular osmotic pressure.

*Cunninghamia lanceolata* seedlings showed different morphological and physiological adjustments in response to reduced light intensity. Morphological adaptations involved photomorphogenetic responses (e.g., height and lateral branch elongation, reduced R∶S, increased H∶D, leaf expansion, and increased SLA), and a resource foraging strategy. Together, these adjustments contributed to enhancement of light interception under the experimental shading conditions. However, low light intensity was not conducive to photosynthesis and carbohydrate synthesis at a high enough rate, as the activities of major carbohydrate-metabolizing enzymes decreased significantly with decreasing light intensity, resulting in reduced accumulation of dry biomass, lower NSC content, and lower seedling QI, especially under the lowest light intensity treatment (5%) tested.

As the primary adaptation strategy to decreasing light intensity, the leaves of *C. lanceolata* seedlings adjusted their physiological parameters, while morphologically, they adopted a biomass allocation pattern consistent with the optimal allocation theory, with a certain degree of self-regulation across a wide range of light intensities. Thus, *C. lanceolata* seedlings self-regulated over a wide range of light intensities, showing morphological and physiological adaptation strategies for photoprotection from high light intensity, and acclimatization and resource foraging under low light intensity. Overall, our findings indicated that light played a major role in determining *C. lanceolata* seedlings morphology and physiology, and showed that *C. lanceolata* is highly vulnerable to shading, and grows best under high sunlight. These findings establish a theoretical foundation for tailoring light optimum light environments in *C. lanceolata* plantations during early seedling growth, thus addressing a critical gap in sustainable forestry management. However, given the potential limitations of our pot experiments (e.g., restricted root growth volume), it is essential to integrate controlled experiments with natural light-gradient surveys to both verify our findings and advance our understanding of the photoresponse by *C. lanceolata* young seedlings, such as to scientifically inform sound forest-management practices.

## Data Availability

The data analyzed in this study is subject to the following licenses/restrictions: Data used in this study will be made available upon reasonable request to the corresponding author. Requests to access these datasets should be directed to Zhijun Huang, fjhuangzj@126.com.
